# Fatty acid elongases 1-3 have distinct roles in mitochondrial function, growth, and lipid homeostasis in *Trypanosoma cruzi*

**DOI:** 10.1016/j.jbc.2023.104715

**Published:** 2023-04-13

**Authors:** Lucas Pagura, Peter C. Dumoulin, Cameron C. Ellis, Maria T. Mendes, Igor L. Estevao, Igor C. Almeida, Barbara A. Burleigh

**Affiliations:** 1 Department of Immunology and Infectious Diseases, Harvard T. H. Chan School of Public Health, Boston, Massachusetts, USA; 2 Department of Biological Sciences, Border Biomedical Research Center, University of Texas at El Paso, El Paso, Texas, USA

**Keywords:** *Trypanosoma cruzi*, fatty acid, lipid synthesis, ELOs, metabolism, growth

## Abstract

Trypanosomatids are a diverse group of uniflagellate protozoan parasites that include globally relevant pathogens such as *Trypanosoma cruzi*, the causative agent of Chagas disease. Trypanosomes lack the fatty acid synthase system typically used for *de novo* fatty acid (FA) synthesis in other eukaryotes. Instead, these microbes have evolved a modular FA elongase (ELO) system comprised of individual ELO enzymes (ELO1-4) that can operate processively to generate long chain- and very long chain-FAs. The importance of ELO’s for maintaining lipid homeostasis in trypanosomatids is currently unclear, given their ability to take up and utilize exogenous FAs for lipid synthesis. To assess ELO function in *T. cruzi,* we generated individual KO lines, Δ*elo1, Δelo2,* and Δ*elo3,* in which the genes encoding ELO1-3 were functionally disrupted in the parasite insect stage (epimastigote). Using unbiased lipidomic and metabolomic analyses, in combination with metabolic tracing and biochemical approaches, we demonstrate that ELO2 and ELO3 are required for global lipid homeostasis, whereas ELO1 is dispensable for this function. Instead, ELO1 activity is needed to sustain mitochondrial activity and normal growth in *T. cruzi* epimastigotes. The cross-talk between microsomal ELO1 and the mitochondrion is a novel finding that, we propose, merits further examination of the trypanosomatid ELO pathway as critical for central metabolism.

The fatty acid (FA) composition of lipid bilayers can profoundly influence the biophysical properties (fluidity, curvature, microdomain organization) and biological functions (secretion, signal transduction, adaptation to stress) of cellular membranes (1, 2, 3). As such, regulation of the FA pools used for synthesis and maintenance of biological membranes is a critical homeostatic function of all cells (4). While these regulatory processes have been well-studied in mammals and yeast (5, 6, 7), the mechanisms governing FA and lipid homeostasis in pathogenic protozoa are poorly understood.

*Trypanosoma cruzi* is a member of a diverse group of flagellated protozoan parasites that include parasitic organisms responsible for globally relevant human diseases such as leishmaniasis (*Leishmania* spp*.*), sleeping sickness (*Trypanosoma brucei*). and Chagas disease (*T. cruzi*). Throughout its complex life cycle, *T. cruzi* colonizes hematophagous triatomine insects and mammalian hosts and switches between actively dividing and nondividing forms in each setting. Survival in these disparate environments requires both morphological and metabolic adaptation (8). A critical feature of adaptation in *T. cruzi* is the ability to alter membrane lipid composition and the fatty acyl moieties of the abundant glycosylphosphatidylinositols that anchor arrays of glycoproteins and glycolipids to the parasite surface (9, 10). The biological consequences of FA remodeling during development are best documented for surface glycosylphosphatidylinositols-anchored mucins, where changes in FA chain length and/or degree of saturation can activate or dampen the proinflammatory activity of these molecules (11). It is currently unknown how *T. cruzi* regulates the composition or abundance of FAs needed for lipid synthesis (12), but similar to other cell types, this protozoan can synthesize long-chain FA (LCFA) endogenously (10, 13) and to take up FAs from the environment (14).

Bulk synthesis of LCFA in most eukaryotes is achieved using a cytosolic type-I fatty acid synthase (15) that is absent in trypanosomatids. Some eukaryotes, such as plants (16, 17, 18) and apicomplexan parasites like *Plasmodium* (19, 20, 21) have instead a type-II fatty acid synthase (FAS-II) system localized in plastids. A mitochondrial FAS-II system is present in trypanosomes (12, 13), which has a more restricted role in cells as a nutrient-sensitive coordinator of mitochondrial oxidative phosphorylation (22) and contributes only about 10% of total FA activity in these parasites (23, 24). Short-chain FA (SCFA) intermediates generated by FAS-II can also be diverted to synthesize other molecules, such as lipoic acid, a critical cofactor needed for the stabilization, and activity of a subset of mitochondrial enzymes (24, 25). Besides having a mitochondrial FAS-II system, trypanosomatids have develop a specialized microsomal FA elongase (ELO) system to synthesize bulk LCFA (10, 13).

First described in *T. brucei* (13), the trypanosomatid ELO system consists of individual FA ELO enzymes (ELOs 1-5 in *T. cruzi* (26)), which exhibit distinct specificities for fatty acyl-CoA substrates based on the carbon-chain length and/or degree of unsaturation (13). ELOs 1-4 are numbered according to their relative position in a sequential pathway (ELO1, ELO2, etc.), where the product of one ELO becomes the substrate for the following enzyme (13). Studies using isolated membranes indicate that the ELO enzymes can also function independently to elongate exogenous fatty acyl substrates of the appropriate chain length (13). ELOs utilize malonyl-CoA as a two-carbon donor to extend fatty acyl-CoA substrates in a four-step cycle involving: (1) a β-ketoacyl-CoA synthase (the “ELO”) responsible for condensing a preexisting fatty acyl-CoA with malonyl-CoA, this is the first and rate-limiting step in each elongation cycle and guides substrate specificity; (2) a β-ketoacyl-CoA reductase; (3) β-hydroxyacyl-CoA dehydratase; and (4) a *trans*-2-enoyl-CoA reductase (10, 13). The ELO1 cycle elongates short-chain fatty acyl-CoA substrates (C4, C6, and C8) to generate C10. Still, unlike type-I fatty acid synthase, ELO1 cannot initiate FA synthesis using acetyl-CoA; instead it uses butyryl-CoA as the main substrate (13). The source of butyryl-CoA for this reaction remains unknown. ELO2 extends C10 and C12 substrates to generate C14 and ELO3 elongates C14 to generate C16 and C18 fatty acids. ELO4 produces very LCFAs (up to 26 carbons in length) (27) and ELO5 acts as a polyunsaturated FA ELO (26). Apart from the initial characterization of the microsomal ELO pathway in *T. brucei* (13) and the demonstration of functional ELOs in *T. cruzi* (13, 26, 28) and *Leishmania* (10, 13, 26), the relative contribution of this pathway toward lipidome maintenance in trypanosomatids has not been determined.

In this study, we examined the role of the ELO system in supporting lipid homeostasis in axenic *T. cruzi* epimastigotes (EPI). As 16- and 18-carbon FA species represent ∼60% of total FA species in this organism (27, 29, 30), we focused our analysis on the ELO pathway enzymes, ELOs 1-3, which are expected to produce C16 and C18 from SCFA substrates in a processive pathway (13). Unbiased lipidomic analyses of loss-of-function mutants, Δ*elo1*, Δ*elo2*, and Δ*elo3,* revealed that both ELO2 and ELO3 contribute significantly to maintaining of global lipidomic profiles in *T. cruzi*. Despite marked lipidome remodeling in Δ*elo2* and Δ*elo3* EPI, lipidomic changes were well-tolerated with little impact on the growth of these mutants in culture. In contrast, ELO1 was found to be dispensable for LCFA synthesis and global lipidome maintenance. Instead, ELO1 expression was required to support mitochondrial metabolism and proliferation. Further analysis revealed that loss of ELO1 function was associated with decreased protein lipoylation, which was rescued by octanoic acid (C8:0) supplementation or genetic complementation with functional *elo1*. Together, these findings highlight the modular nature of the trypanosomatid ELO pathway and reveal an unexpected role for ELO1 in supporting aspects of mitochondrial metabolism in *T. cruzi* EPI, including impact on the generation of SCFA precursors for lipoic acid synthesis, a function generally attributed to mitochondrial FAS-II.

## Results

### Generation of ELO-deficient *T. cruzi* mutants

**Figure 1:**
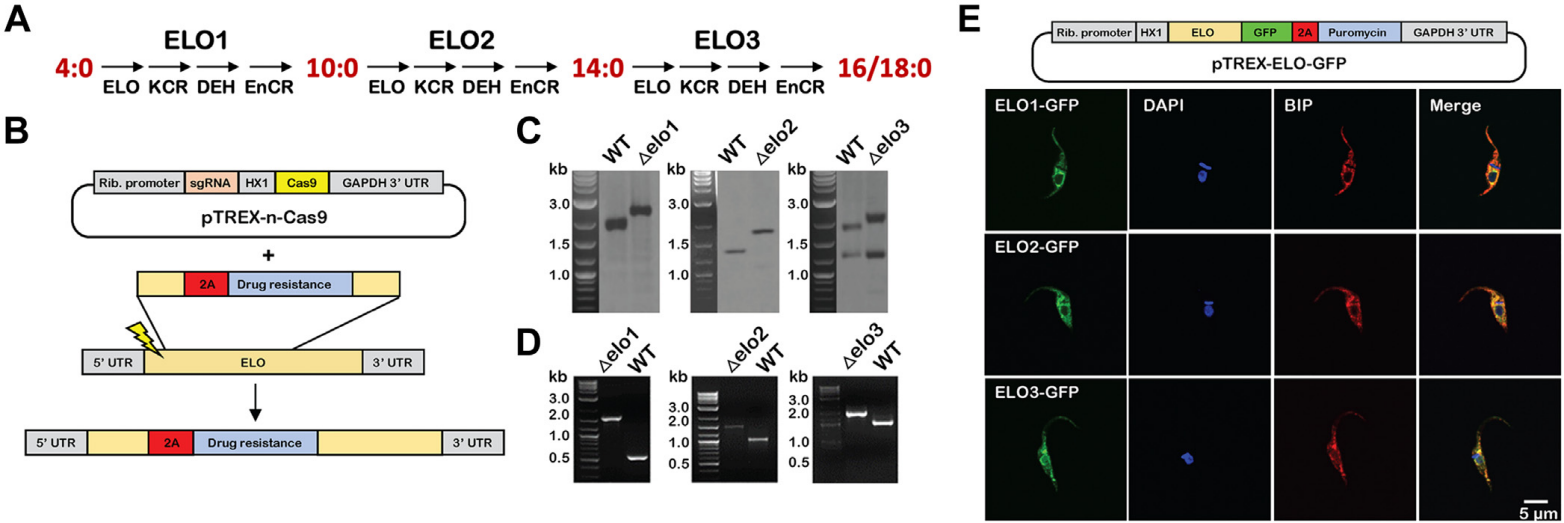
**Generation of ELO-deficient and genetically complemented *T. cruzi* lines.***A*, schematic of ELO pathway with primary FA substrates and products shown (*red*). *B*, general strategy for the targeted disruption of genomic loci in *T. cruzi* EPI using a CRISPR/Cas9-mediated homology-directed repair approach. *C*, Southern blots of digested genomic DNA isolated from WT or different *T. cruzi elo* mutants and (*D*) gDNA PCRs confirm the integration of KO constructs at the correct locus. *E*, schematic of pTREX expression vector used for genetic complementation of Δ*elo* mutants. Expression of GFP-tagged ELOs in genetically complemented Δ*elo* mutant epimastigotes reveals an ELO-GFP (*green*) signal overlap with the endoplasmic reticulum chaperone BiP (*red*). Parasite nuclear and kinetoplast DNA were visualized using DAPI (*blue*). DEH, hydroxyacyl-CoA dehydratase; EnCR, *trans*-2-enoyl-CoA reductase; FA, fatty acid; KCR, ketoacyl-CoA reductase.

The *T. cruzi* FA ELO genes, *elo1* (TcCLB.506661.30/TcCLB.511245.130) *elo2* (TcCLB.506661.20/TcCLB.511245.140), and *elo3* (TcCLB.506661.10/TcCLB.511245.150) (Fig. 1*A*), were individually targeted for disruption in axenic *T. cruzi* EPI using CRISPR/Cas9 and integration of homology-directed repair cassettes (Fig. 1*B*). Cloning of transfected EPI during initial drug selection post-transfection facilitated recovery of parasites with insertions in both alleles with the successful generation of three independent ELO KOs, Δ*elo1*, Δ*elo2*, and Δ*elo3*, as confirmed by Southern blot (Fig. 1*C*) and PCR (Fig. 1*D*). Genetic complementation of each Δ*elo* mutant was achieved with a modified pTREX expression vector encoding a C-terminal GFP-tagged copy of the relevant full-length *elo* gene on the mutant background (Fig. 1*E*). Consistent with the reported microsomal localization of the ELO pathway enzymes in *T. brucei* (13), all ELO-GFP proteins localized to the endoplasmic reticulum (ER) in *T. cruzi* EPI as confirmed by colocalization with the ER chaperone BiP (31) (Fig. 1*E*).

### Lipidomic characterization of *T. cruzi* ELO mutants

**Figure 2:**
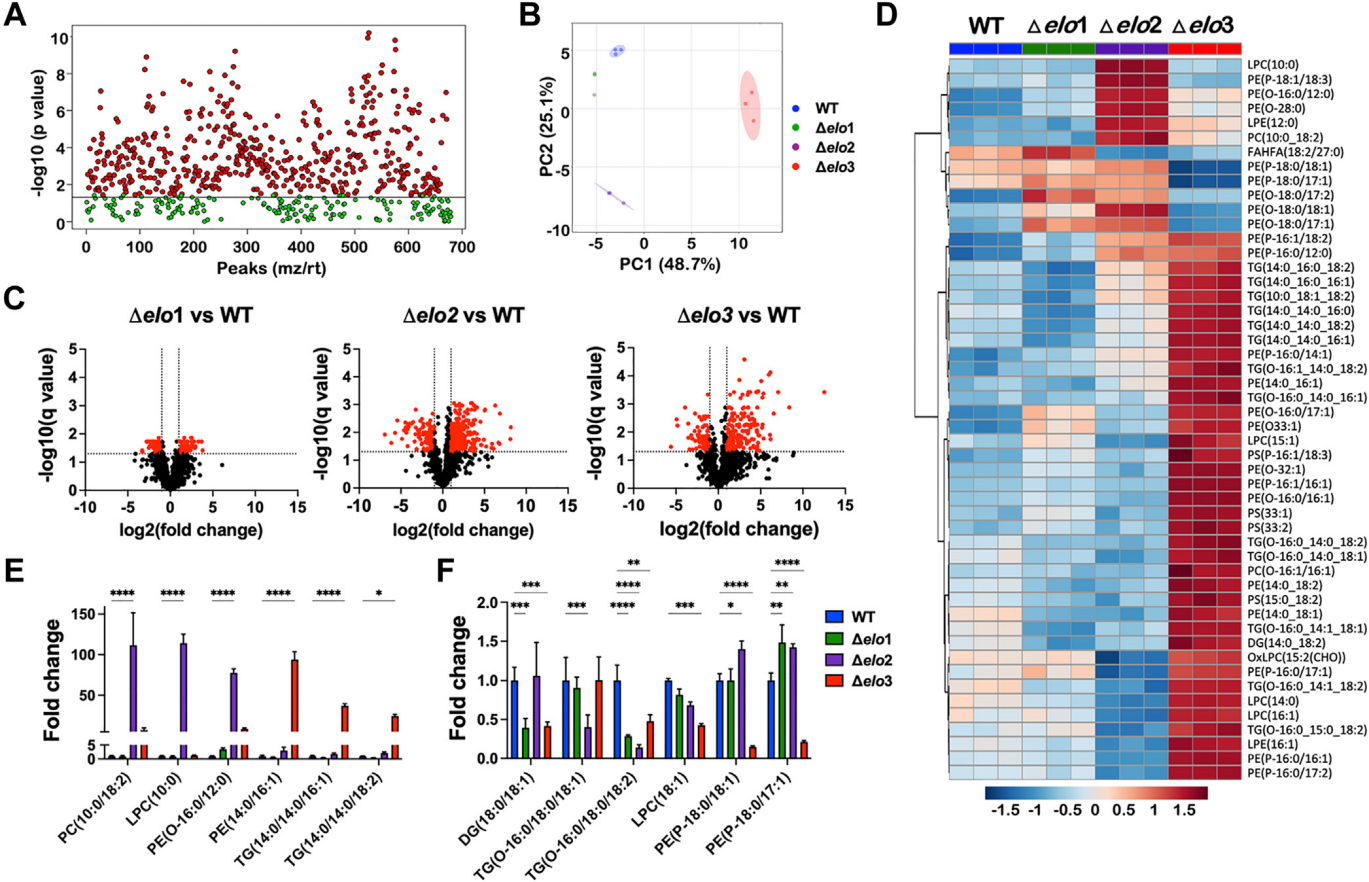
**Lipidomic characterization of *T. cruzi* Δ*elo* mutants.** Lipids identified in WT and Δ*elo* mutant *T. cruzi* epimastigotes (Folch’s lower phase) using UHPLC-HR-LC-MS/MS. *A*, *one-way ANOVA* with Tukey’s posthoc analysis showing significant differences in abundance (*p* < 0.05). *B*, principal component analysis (PCA) of total lipidomes plotted for WT, Δ*elo*1, Δ*elo*2, and Δ*elo*3 *T. cruzi* EPI. The first two principal components are shown (PC1 and PC2) with the proportion of variance for each component in parentheses. Biological replicates for each experimental group are represented and the 95% confidence interval is indicated in a *shaded circle*. *C*, volcano plots based on fold-change (*x*-axis) and adjusted *p*-value (q-value; *y*-axis) reveal differences in lipid profiles. The *horizontal line* indicates a significant q value (q ≤ 0.05), and *vertical lines* indicate ≥2-fold change-≤0.5 cut-off. Lipids that are significantly altered in abundance (≥2-fold change or ≤0.5-fold change; adjusted *p*-value ≤ 0.05) are represented as *red dots* in each pair-wise comparison. *D*, heatmap displaying the 50 most significantly upregulated or downregulated lipids when comparing Δ*elo* mutants with WT EPI. Highlighted examples of lipids in the dataset that are significantly increased (*E*) or decreased (*F*) in one or more Δ*elo* mutant(s) as compared to *T. cruzi* WT EPI are shown. Two-way ANOVA with Tukey’s multiple comparisons test was applied (∗*p* < 0.05, ∗∗*p* < 0.01, ∗∗∗*p* < 0.001, ∗∗∗∗*p* < 0.0001). Cer, ceramides; DG, diacylglycerol; FAHFA, fatty acyl esters of hydroxy fatty acids; LPC, *lyso*-phosphatidylcholine; LPE, *lyso*-phosphatidylethanolamine; PC, phosphatidylcholine; PE, phosphatidylethanolamine; PI, phosphatidylinositol; PS, phosphatidylserine; TG, triacylglycerol; UHPLC, ultra-HPLC.

**Supporting Figure S1:**
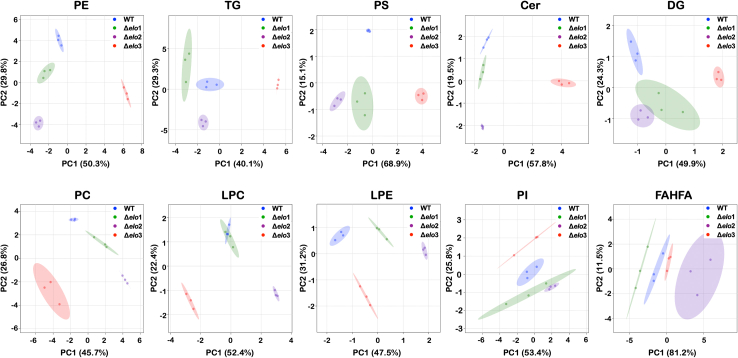
**PCA plots of WT and Δ*elo* mutants by lipid class.** Principal component analysis of lipid species plotted for the indicated lipid subclasses: PE, phosphatidylethanolamine; TG, triacylglycerol; PS, phosphatidylserine; Cer, ceramides; DG, diacylglycerol; PC, phosphatidylcholine; LPC, *lyso*-phosphatidylcholine; LPE, *lyso*-phosphatidylethanolamine; PI, phosphatidylinositol; FAHFA, fatty acyl esters of hydroxy fatty acids. The first two principal components are plotted (PC1 and PC2) with the proportion of variance for each component in parenthesis and the 95% confidence interval indicated in *shaded areas*.

As the main system used for bulk LCFA synthesis in trypanosomatids, the ELO pathway is assumed to play a critical role in membrane lipid synthesis and remodeling in this group of organisms. Still, the contribution of this pathway in shaping the lipidome in these organisms has yet to be determined experimentally. To evaluate the impact of disruption of ELO pathway enzymes on the lipid composition of *T. cruzi* parasites, we performed label-free, quantitative ultra-HPLC (UHPLC) coupled to high-resolution tandem MS (UHPLC-HR-MS/MS) of lipids extracted from WT, Δ*elo1*, Δ*elo2*, Δ*elo3* and genetically complemented EPI lines. A total of 1133 lipid species were identified after manual curation of LipidSearch-assigned IDs (ThermoFisher) (Table S1), the majority of which were found to be significantly altered in abundance (*p* < 0.05; one-way ANOVA) across all mutants as compared to WT EPI (Fig. 2*A*). Two-dimensional principal component analysis (PCA) identified overall trends in the lipidome data (Fig. 2*B*), where the Δ*elo3* mutant emerged as the distinct outlier, well-separated from the other parasite lines along both PC1 and PC2 (Fig. 2*B*). The Δ*elo1* EPI lipidome was the least divergent from WT and Δ*elo*2 separated from both of these lines along PC2 (Fig. 2*B*). When broken down according to major lipid subclass (Fig. S1) PCA clustering patterns were found to be similar to that observed for the total lipidome (Fig. 2*B*), indicating that global lipidomic differences between of WT and Δ*elo* mutant *T. cruzi* EPI are likely not driven by specific lipid subclasses. Volcano plots displaying pairwise comparisons of WT and individual Δ*elo* mutants (Fig. 2*C*) and heatmap data highlighting the 50 lipids that changed most dramatically in the mutants compared to WT EPI (Fig. 2*D*) demonstrate that lipidomic changes are most prominent in Δ*elo2* and Δ*elo3* mutants. Consistent with this conclusion, 232 and 149 lipids were found to be changed ≥2- or ≤0.5-fold change (*p*-value < 0.05) in Δ*elo2* and Δ*elo3*, respectively (Table S2). In contrast, comparatively few lipids (48) exhibited ≥2- or ≤0.5-fold change in abundance in the Δ*elo1* mutant when compared to WT EPI (Fig. 2, *C* and *D*) (Table S2).

**Supporting Figure S2:**
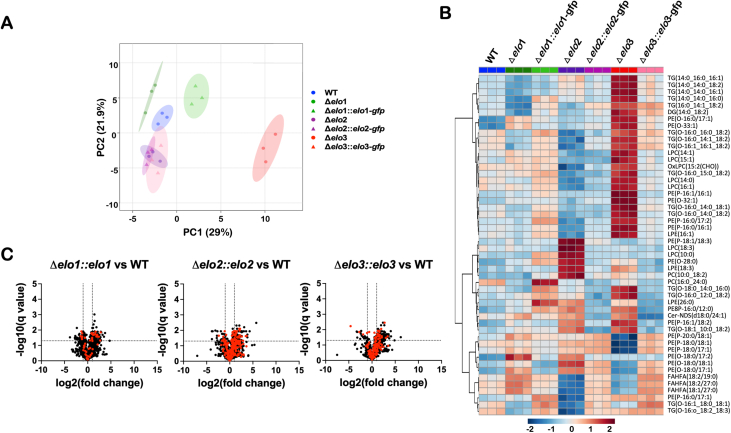
**Lipidomic characterization of *T. cruzi* Δ*elo* mutants and genetically complemented lines.***A*, Principal component analysis (PCA) of total lipidome data plotted for WT, each Δ*elo* mutant and corresponding genetically-complemented mutant *T. cruzi* EPI. The first two principal components are shown (PC1 and PC2) with the proportion of variance for each component in parenthesis. Biological replicates for each experimental group are represented and the 95% confidence interval indicated in *shaded circle*. *B*, Heat map displaying the 50 most significantly up or down regulated lipids when comparing Δ*elo* mutants and genetically-complemented *T. cruzi* EPI with WT parasites. *C*, Volcano plots generated based on fold-change (x-axis) and adjusted *p*-value (q value; y-axis) in pairwise comparisons between WT and genetically-complemented lines: Δ*elo1::elo1-gfp,* Δ*elo2::elo2-gfp,* and Δ*elo3::elo3-gfp T. cruzi* EPI. *Horizontal line* represent significant q value (q ≤ 0.05), *vertical lines* indicate ≥2-fold change-≤0.5 cut-off. Lipids found to be significantly altered in abundance (≥2-fold change or fold change-≤0.5; adjusted *p*-value ≤ 0.05) in the corresponding Δ*elo* mutant (Fig. 2*C*) are highlighted (*red circles*) for each comparison. PE, phosphatidylethanolamine; TG, triacylglycerol; PS, phosphatidylserine; Cer, ceramides; DG, diacylglycerol; PC, phosphatidylcholine; LPC, *lyso*-phosphatidylcholine; LPE, *lyso*-phosphatidylethanolamine; PI, phosphatidylinositol; FAHFA, fatty acyl esters of hydroxy fatty acids.

Consistent with the anticipated function of ELO3 in extending 14-carbon FAs to C16 and C18 species, we noted a marked reduction in C18:0- and C18:1-containing lipids in Δ*elo*3 EPI and a concomitant increase in lipids containing C14:0, C14:1, C16:0, and C16:1 in this mutant (Fig. 2, *D*–*F*). A similar trend was observed for Δ*elo*2 EPI, where lipids containing C10:0 and C12:0 were elevated, and lipids containing C14:0 and C16:0 FA were present at decreased levels (Fig. 2, *D*–*F*). Given that most lipid alterations observed in the Δ*elo* mutants were restored in the genetically complemented lines (Fig. S2), we conclude that the observed lipidomic changes are due to the loss of specific ELO enzyme function. Combined, these data provide the first demonstration that ELO2 and ELO3 play important roles in lipid in *T. cruzi* but that ELO1 contributes little to LCFA synthesis and global lipidomic profiles in this organism.

### Metabolic labeling and FA substrate elongation in WT and Δelo mutants

**Figure 3:**
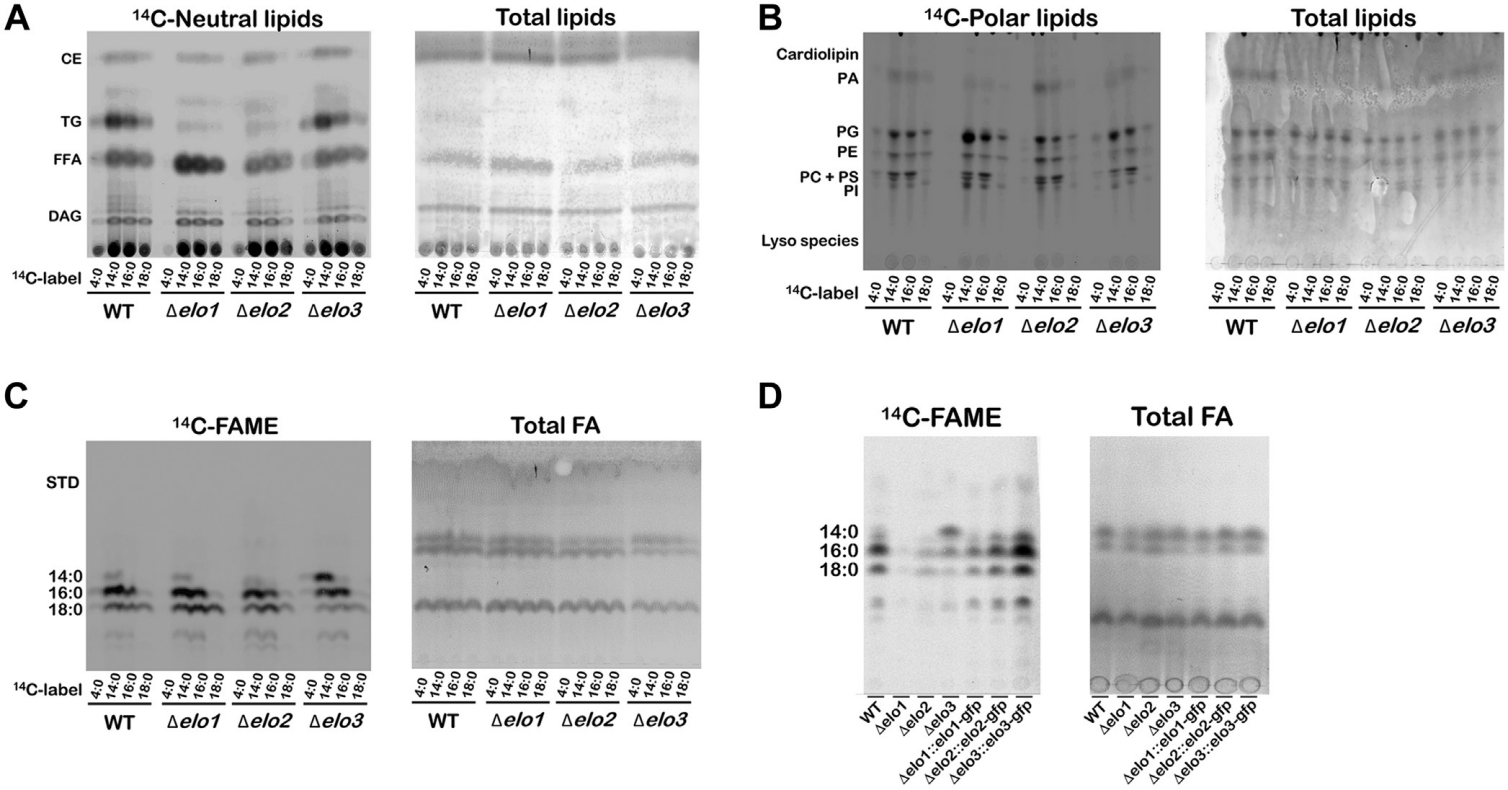
**Elongation and utilization of exogenous fatty acid substrates by *T. cruzi* WT and Δ*elo* mutants.** Metabolic tracing showing exogenous FA incorporation and elongation in WT and Δ*elo* mutants by TLC. Exponentially growing epimastigotes were incubated with 0.3 μCi/ml of different 1-^14^C-FA, as indicated (^14^C-label) for 18 h before to lipid extraction. *Left and right panels* show ^14^C-labeled lipids and total lipids (sulfuric acid-copper staining), respectively. Neutral and polar lipids (*A* and *B*, respectively) were isolated from 1-^14^C-FA-labeled *T. cruzi* EPI, resolved by RP-TLC and exposed to film for 3 days. *C*, fatty acid methyl esters (FAME) derived from 1-^14^C-FA-labeled lipids resolved by RP-TLC; film exposed for 3 days. *D*, ^14^C-FAME resulting from the labeling of *T. cruzi* WT, Δ*elo* mutants and genetically complemented lines with ^14^C-butyric acid, resolved by RP-TLC and exposed to film for 10 days. CE, cholesterol ester; DAG, diacylglycerol; FA, fatty acid; FFA, free fatty acid; PA, phosphatidic acid; PC, phosphatidylcholine; PE, phosphatidylethanolamine; PG, phosphatidylglycerol; PI, phosphatidylinositol; PS, phosphatidylserine; RP, reversed phase; TG, triacylglycerol.

In addition to *de novo* synthesis of FAs, *T. cruzi* takes up FA and lipids from the environment that can be incorporated into their own lipid pools (14, 32, 33, 34, 35). The observation that ELO2- and ELO3-deficient EPI experience global lipidomic changes, characterized by a decrease in lipids containing 18-carbon FA side chains, despite continuous culture in medium containing 10% serum, suggests that serum lipids/exogenous FAs are insufficient to overcome the loss of ELO activity. To examine the possibility that *T. cruzi* Δ*elo* mutants may experience defects in their ability to take up or utilize exogenous FA for lipid synthesis, we performed metabolic tracing studies using [1-14C]-labeled FAs of varying chain lengths (C4:0, C14:0, C16:0, and C18:0). Lipids extracted from ^14^C-FA-labeled parasites were resolved using reversed-phase (RP) TLC to identify major lipid classes into which label was incorporated (Fig. 3, *A* and *B*). Although differences in ^14^C-label labeling intensity were observed with the different ^14^C-FA provided, label was incorporated into neutral (Fig. 3*A*) and polar (Fig. 3*B*) lipids across all parasite lines (Fig. 3, *A* and *B*). A notable exception was the Δ*elo1* mutant, which failed to incorporate 14C-butyric acid (C4:0) carbons into parasite neutral and polar lipids (Fig. 3, *A* and *B*).

To more directly assess the impact of specific ELO-deficiencies on FA elongation in intact parasites, FA methyl esters (FAMEs) generated from total lipids extracted from each parasite line following ^14^C-FA labeling were resolved by TLC (Fig. 3*C*). As expected, carbons from exogenous [1-14C]-FA were detected in LCFA (C14:0, C16:0, and C18:0) in all parasite lines, except for Δ*elo1*, which failed to significantly incorporate ^14^C from labeled butyric acid into LCFA species (Fig. 3*C*). Given the relatively faint signal in WT parasites obtained when ^14^C-butyric acid was used as a metabolic tracer (Fig. 3, *A*–*C*), additional labeling experiments were performed to optimize the detection of labeled lipids following incubation with ^14^C-butyric acid (Fig. 3*D*). Results show significantly reduced incorporation of ^14^C from butyric acid into LCFAs that is unique to the Δ*elo1* mutant but was fully restored in genetically complemented Δ*elo1::elo1*-*gfp* parasites (Fig. 3*D*). Faint bands for Δ*elo1* mutant, suggest that some butyric acid is being elongated presumably by a less efficient activity of ELO2. Given that Δ*elo1* EPIs readily elongate ^14^C-myristic acid (C14:0) and ^14^C-palmitic acid (C16:0) substrates (Fig. 3*C*), it is unlikely that impaired import of FAs explains the inability of this mutant to elongate ^14^C-butyric acid. Together, these results confirm the function of *T. cruzi* ELO1 as a SCFA ELO that utilizes butyric acid (butyryl-CoA) as a substrate for elongation (13). Moreover, the unimpeded ability of Δ*elo1* EPI to elongate C14:0 and C16:0 substrates, presumably through the actions of ELO2 and ELO3, is consistent with a modular ELO system, in which individual ELOs can function independently of the other pathway enzymes (13). It is worth noting the reduced labeling of neutral and polar lipids Fig. 3, *A* and *B*) and FAMEs (Fig. 3, *C* and *D*) in the Δ*elo2* mutant when ^14^C-butyric acid was provided as substrate. These findings indicate that the products of ELO1 (≤10 carbons length), which are generally not found in the neutral and polar lipids of *T. cruzi* EPI (Table S1), need to be elongated further, before incorporation into these lipids. Given that carbons from ^14^C-butyric acid were detectable in polar lipids (Fig. 3*B*) and C16, C18 FAMEs (Fig. 3*D*) in the Δ*elo2* mutant, albeit at a lower intensity than in WT or Δ*elo3* EPI, point to partial redundancy in the system, possibly through extended capabilities of ELO1 and/or ELO3. Functional overlap and redundancy within the ELO pathway may explain why disruption of ELO2 or ELO3, individually, fails to promote more profound lipidome perturbation in the KO parasite lines.

### ELO1 deficiency is associated with impaired growth of *T. cruzi* epimastigotes

**Figure 4:**
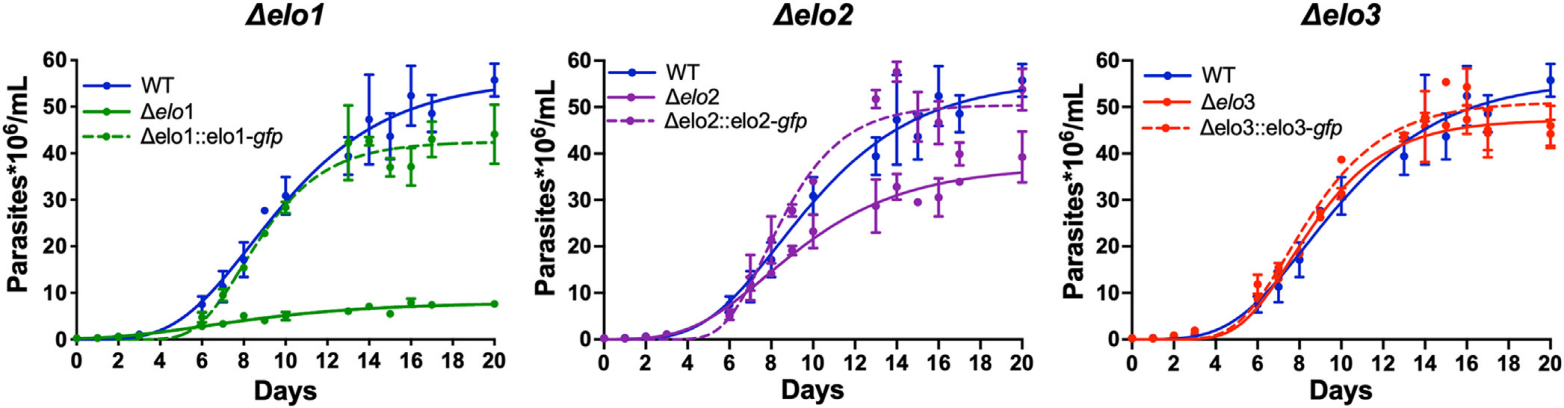
**Growth characteristics of *T. cruzi* Δ*elo* mutants and complemented lines.** The growth of WT, Δ*elo* mutants, and genetically complemented *T. cruzi* EPI lines in LIT medium containing 10% fetal bovine serum was followed for 20 days. The growth of each Δ*elo* mutant and cognate genetically complemented line compared to WT EPI is shown on separate graphs. The mean and SD calculated for parasite density (parasites × 10^6^/ml medium; *y*-axis) for three biological replicates against time (days; *x*-axis) is plotted. LIT, liver infusion tryptose.

**Supporting Figure S3:**
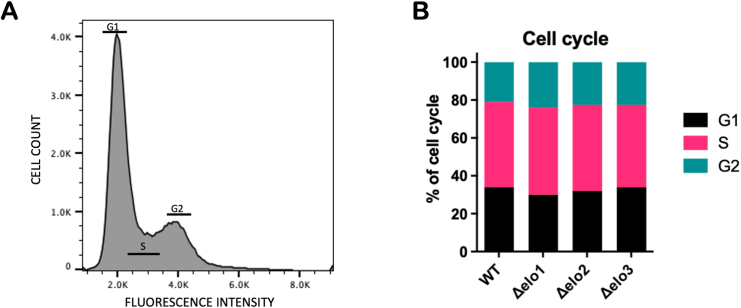
**Cell cycle analysis of *T. cruzi* EPI.** Flow cytometry was used to assess the cycle-cycle status of log-phase *T. cruzi* EPI populations. *A*, Representative histogram obtained following flow cytometry of DAPI-stained *T. cruzi* EPI showing the number of cells in each cell-cycle stage, inferred by measuring the DNA content (DAPI staining). *B*, Percentage of parasites in the G1, S and G2 stages of the cell- cycle.

**Supporting Figure S4:**
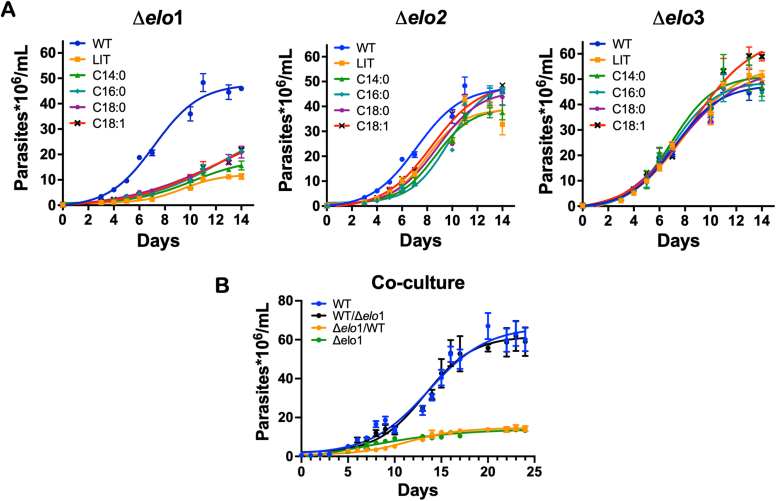
**Growth curves in supplemented or conditioned media.***A*, LCFA supplementation has minimal impact on *T. cruzi* epimastigote growth. The growth of Δ*elo* mutant *T. cruzi* EPI lines in LIT medium containing 10% FBS, with or without supplementation with 35 μM of indicated fatty acid were compared to WT EPI in LIT medium + 10% FBS and shown on separate graphs. LIT: LIT + 10% FBS; no FA added. C14:0; myristic acid, C16:0; palmitic acid, C18:0; stearic acid, C18:1, oleic acid. *B*, Growth curves of WT and Δ*elo1* mutant in transwell plates with a 0.4 μm pore size to allow media exchange. WT/Δ*elo1* refers to WT parasites grown in the transwell plate. Δ*elo1*/WT refers to Δelo*1* parasites grown in the transwell plate. The mean and s.d. calculated for parasite density (parasites × 10^6^/ml medium; y-axis) for three biological replicates against time (days; x-axis) is plotted.

To evaluate the biological impact of ELO gene disruption in *T. cruzi*, we first examined the growth kinetics of Δ*elo1*, Δ*elo2*, and Δ*elo3* EPI in liver infusion tryptose (LIT) medium containing 10% serum (Fig. 4). Strikingly, ELO1-deficient *T. cruzi* EPI exhibit marked growth impairment in the log-phase (doubling time [DT] of 60 ± 12 h) compared to WT parasites (DT = 31 ± 7 h). Δ*elo2* EPIs exhibit a mild growth defect (DT = 41 ± 9 h), whereas the Δ*elo3* mutant replicates at rates similar to WT (Fig. 4). The reduced rates of growth observed for Δ*elo1* and Δ*elo2* EPIs do not involve changes in the parasite cell cycle, as the proportion of parasites associated with different phases of the cell cycle (G1-S-G2/M) remained unaltered in each mutant as compared to WT (Fig. S3). The growth deficits observed for Δ*elo1* and Δ*elo2* mutants were rescued following stable expression of ELO1-GFP or ELO2-GFP in the respective mutant lines (Fig. 4). As expected, growth of genetically complemented Δ*elo3* parasites exhibited similar kinetics as WT and Δ*elo3* EPI (Fig. 4). In contrast to genetic complementation, attempts to rescue growth of Δ*elo1* or Δ*elo2* EPI by supplementation of the medium with exogenous FAs of varying chain lengths (14:0, 16:0, 18:0, or 18:1) failed to fully restore growth of these mutants, despite mild beneficial effects of supplemental C18 FAs on parasite growth (Fig. S4*A*). To investigate whether changes in the growth medium, including secretion/release of parasite derived factors, could contribute to the slowed growth of ELO1-deficient EPI, the Δ*elo1* mutant was cocultured with WT EPI in transwell microplates, separated by a 0.4 μm pore membrane, to allow exchange of medium and parasite-derived factors including metabolites and shed membranous vesicles between the two compartments. These conditions failed to improve Δ*elo1* EPI growth with no discernable impact on WT EPI growth (Fig. S4*B*), indicating that any alterations in the extracellular environment are not responsible for the growth phenotypes observed.

Overall, our data demonstrate that when targeted individually, ELO1, ELO2, and ELO3 are dispensable for *T. cruzi* EPI growth and that the extent of lipidome perturbation resulting from loss of ELO activity correlates poorly with growth phenotypes in the Δ*elo* mutants. Thus, loss of ELO1 function may promote specific changes in the parasite lipidome not captured in our analysis and/or other metabolic changes that lead to growth impairment in Δ*elo1* and, to a lesser extent, Δ*elo2* EPI.

### ELO1 deficiency leads to metabolic changes and mitochondrial dysfunction

**Figure 5:**
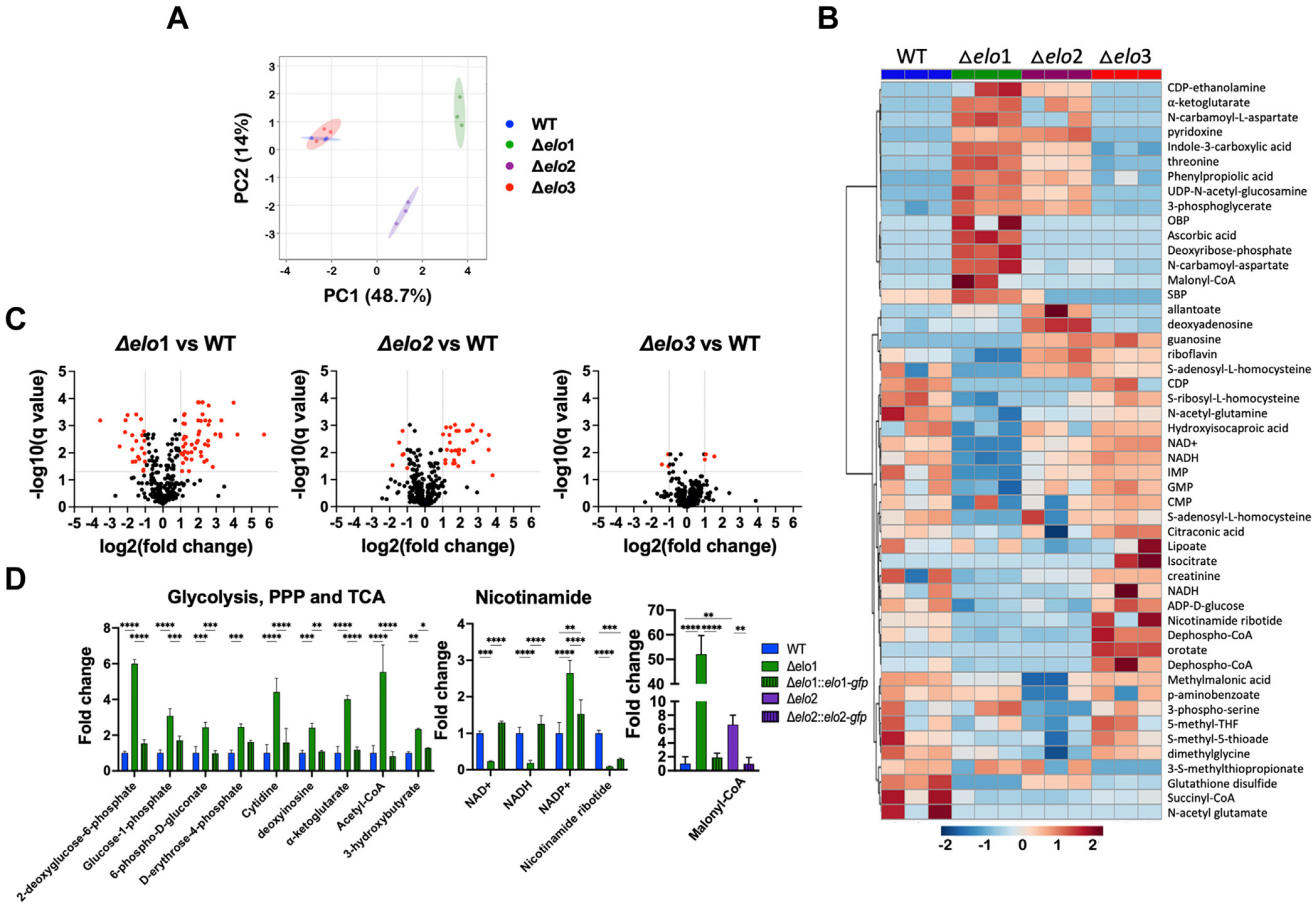
**Metabolite perturbation in Δ*elo* mutant *T. cruzi* EPI.** PCA scores plot (*A*) and heat map (*B*) comparing steady-state metabolite profiles of *T. cruzi* WT and Δelo mutant EPI. *C*, volcano plots based on fold-change (*x*-axis) and adjusted *p*-value (q value; *y*-axis). The *horizontal line* represents a significant q value (q ≤ 0.05), and *vertical lines* indicate ≥2- and ≤0.5-fold change cutoffs. Highlighted metabolites (*red circles*) are significantly altered in abundance (≥2- or ≤0.5-fold change; adjusted *p*-value < 0.05) in pair-wise comparisons of WT EPI and individual Δ*elo* mutant strains. *D*, relative abundance of metabolites shown to be significantly altered in Δ*elo1* mutant EPI and restored to WT levels in Δ*elo1::elo1-gfp*. Two-way ANOVA with Tukey’s multiple comparisons test was applied (∗*p* < 0.05, ∗∗*p* < 0.01, ∗∗∗*p* < 0.001, ∗∗∗∗*p* < 0.0001). Results show metabolites from different metabolic pathways significantly changing in Δ*elo*1 and, to a lesser extent, in Δ*elo*2 EPI. PCA, principal component analysis.

**Supporting Figure S5:**
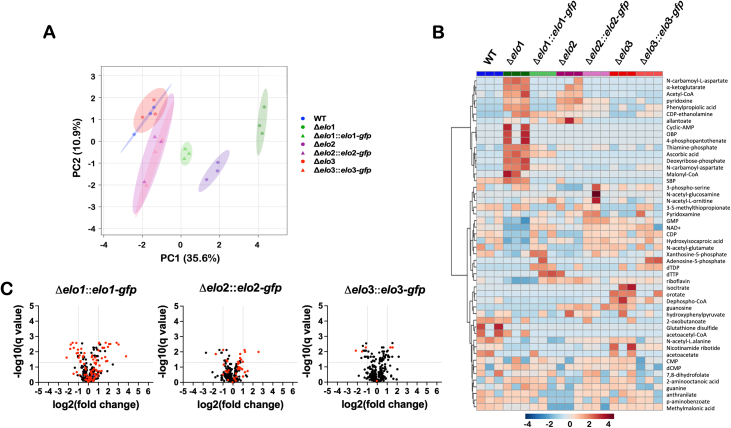
**Metabolomic analysis of WT, Δ*elo* mutants and genetically complemented strains.***A*, Principal component analysis (PCA) plot of metabolite data plotted for WT, each Δ*elo* mutant and corresponding genetically-complemented mutant *T. cruzi* lines. The first two principal components are shown (PC1 and PC2) with the proportion of variance for each component in parenthesis. Biological replicates for each experimental group are represented and the 95% confidence interval indicated in shaded circle. *B*, Heat map displaying the 50 metabolites displaying the greatest change in parasite groups as compared to WT *T. cruzi* EPI. *C*, Volcano plots generated based on fold-change (x-axis) and adjusted *p*-value (q value; y-axis) in pairwise comparisons between WT and genetically-complemented lines: Δ*elo1::elo1-gfp,* Δ*elo2::elo2-gfp,* and Δ*elo3::elo3-gfp T. cruzi* EPI. *Horizontal lines* represent significant q value (q ≤ 0.05), *vertical lines* indicate ≥2-fold change-≤0.5 cut-off. Metabolites found to significantly altered in abundance (≥2-fold change or fold change-≤0.5; adjusted *p*-value <0.05) in each corresponding Δ*elo* mutant (Fig. 5*C*) is highlighted (*red circles*) for each comparison.

To examine whether disruption of specific FA ELOs in *T. cruzi* triggered other metabolic changes in the Δ*elo* mutant that might explain observed growth phenotypes, we performed an unbiased metabolomic analysis of log-phase WT EPI, each Δ*elo* mutant and the respective genetically complemented lines. LC-MS/MS analysis identified ∼300 metabolites with high confidence in *T. cruzi* EPI extracts (Table S3). PCA allowed visualization of the overall trend in the metabolite data, where WT and Δ*elo3* EPI clustered together, and the Δ*elo2* and Δ*elo1* mutants formed distinct clusters that were well-separated from WT (Fig. 5*A*). These trends were reflected in the heatmap generated with the top 50 metabolites found to be altered in abundance when Δ*elo* mutants were compared to WT *T. cruzi* EPI (Fig. 5*B*) and visualized as pair-wise comparisons in volcano plots (Fig. 5*C*). Among the mutants, Δ*elo1* exhibited the greatest degree of metabolic perturbation with significant changes occurring for 74 metabolites (≥2- or ≤0.5-fold change; q-value < 0.05), 48 of which returned to WT levels in Δ*elo1::elo1-gfp* EPI (Fig. S5). These changes included metabolites associated with glucose metabolism, the pentose–phosphate pathway, nicotinamide synthesis, and the citric acid cycle (Fig. 5*D* and Table S4). By comparison, 37 metabolites were significantly altered in abundance in Δ*elo2* EPI, with the majority restored to WT levels in Δ*elo2::elo2-gfp* parasites. In contrast, the Δ*elo3* mutant showed little evidence of metabolic perturbation, with only six metabolites exhibiting significant changes in abundance as compared to WT. Collectively, these data suggest that metabolite perturbation, and not global lipidomic alterations, correlate with reduced growth phenotypes in Δ*elo1* EPI. Elevation of specific metabolites such as 2-deoxyglucose-6-phosphate (6-fold) and malonyl-CoA (50-fold) in Δ*elo1* EPI (Fig. 5*D* and Table S4), which has the potential to inhibit glycolysis and FA oxidation (FAO), respectively, may be indicative of dysregulated energy metabolism in this mutant.

**Figure 6:**
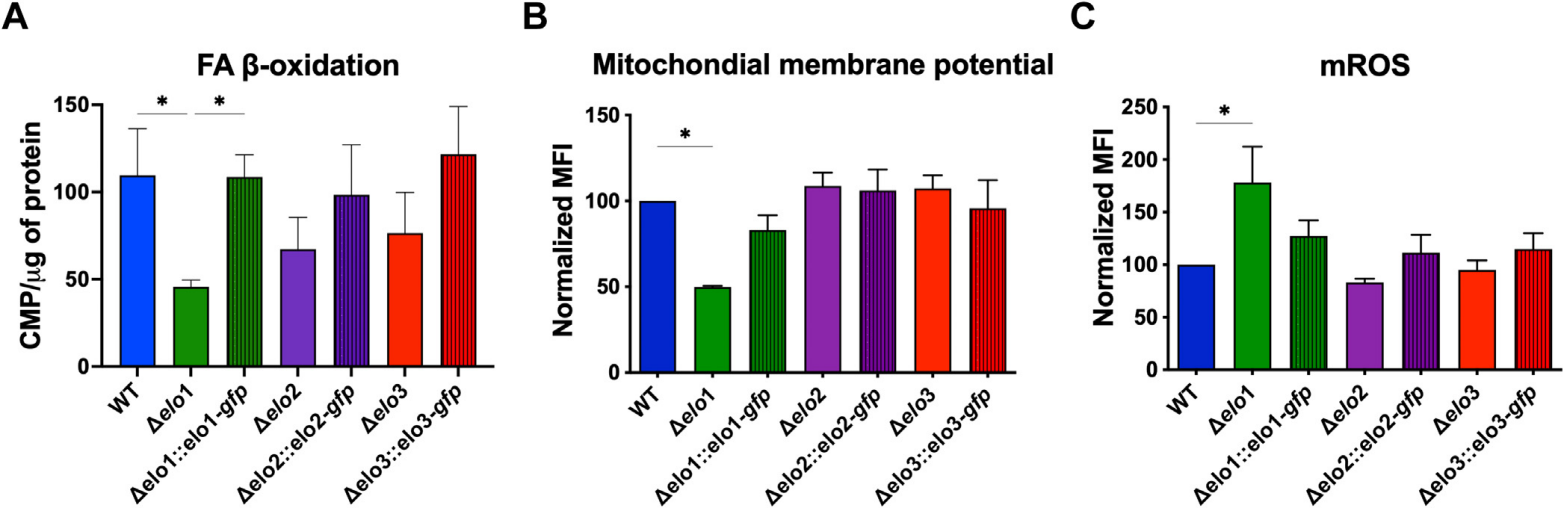
**Fatty acid β-oxidation and mitochondrial function.***A*, fatty acid oxidation was measured in all *T. cruzi* EPI lines following the detection of ^3^H_2_0 released from parasites into the culture supernatant after a 4 h incubation of log-phase EPI with ^3^H-palmitate. Counts were acquired for 5 min in a scintillation counter and normalized to μg of protein in the pelleted parasites. *B*, mitochondrial membrane potential measured in *T. cruzi* EPI lines by TMRE staining and flow cytometric analysis. *C*, mitochondrial reactive oxygen species measured by MitoSOX staining and analyzed by flow cytometry. One-way ANOVA with Dunnett's multiple comparisons test (∗*p* < 0.05) was used for all statistical analyses.

Malonyl-CoA is critical in regulating FA synthesis and oxidation (36). It is generated from acetyl-CoA and is the 2-carbon donor used in the synthesis/elongation of FAs. Malonyl-CoA also inhibits FAO in the mitochondria by blocking the carnitine palmitoyltransferase 1-dependent import of LCFA into the mitochondria (37, 38). To determine if FAO is impaired in *T. cruzi* Δ*elo* mutants, log-phase EPIs were incubated for 4 h with ^3^H-palmitate (C16:0) without glucose, followed by the measurement of ^3^H_2_O released into the supernatant. We observed a significant reduction of FAO in Δ*elo1* EPI that was restored in the genetically complemented line. In contrast, no significant differences were observed in FAO in Δ*elo2* and Δ*elo3* mutants compared to WT EPI (Fig. 6*A*).

Reduced FAO and changes in the abundance of metabolites involved in nicotinamide and NAD synthesis (Fig. 5*D*) led us to ask if mitochondrial function might be affected in the Δ*elo1* mutant. As general indicators of mitochondrial fitness, we measured mitochondrial membrane potential and mitochondrial reactive oxygen species (mROS). We found that both measures are altered in the Δ*elo1* mutant (Fig. 6, *B* and *C*). Mitochondrial membrane potential was reduced in Δ*elo1* mutant by ∼50% compared to WT parasites. No significant changes in membrane potential were observed in Δ*elo2* or Δ*elo3* EPI (Fig. 6*B*). Additionally, mROS levels were differentially elevated in Δ*elo1* EPI (Fig. 6*C*) and both membrane potential and mROS levels restored to WT levels in genetically complemented Δ*elo1::elo1-gfp* parasites (Fig. 6, *B* and *C*). Thus, loss of ELO1 function leads to mitochondrial dysfunction in *T. cruzi* EPI. In contrast, the metabolic and/or lipidomic changes accompanying the loss of ELO2 or ELO3 do not impair mitochondrial function. Reduced mitochondrial membrane potential in Δ*elo1* EPI could indicate reduced electron transport chain activity, possibly through the depletion of NADH and FADH2 produced in the tricarboxylic acid (TCA) cycle and during FAO.

### ELO1 deficiency affects protein lipoylation

**Figure 7:**
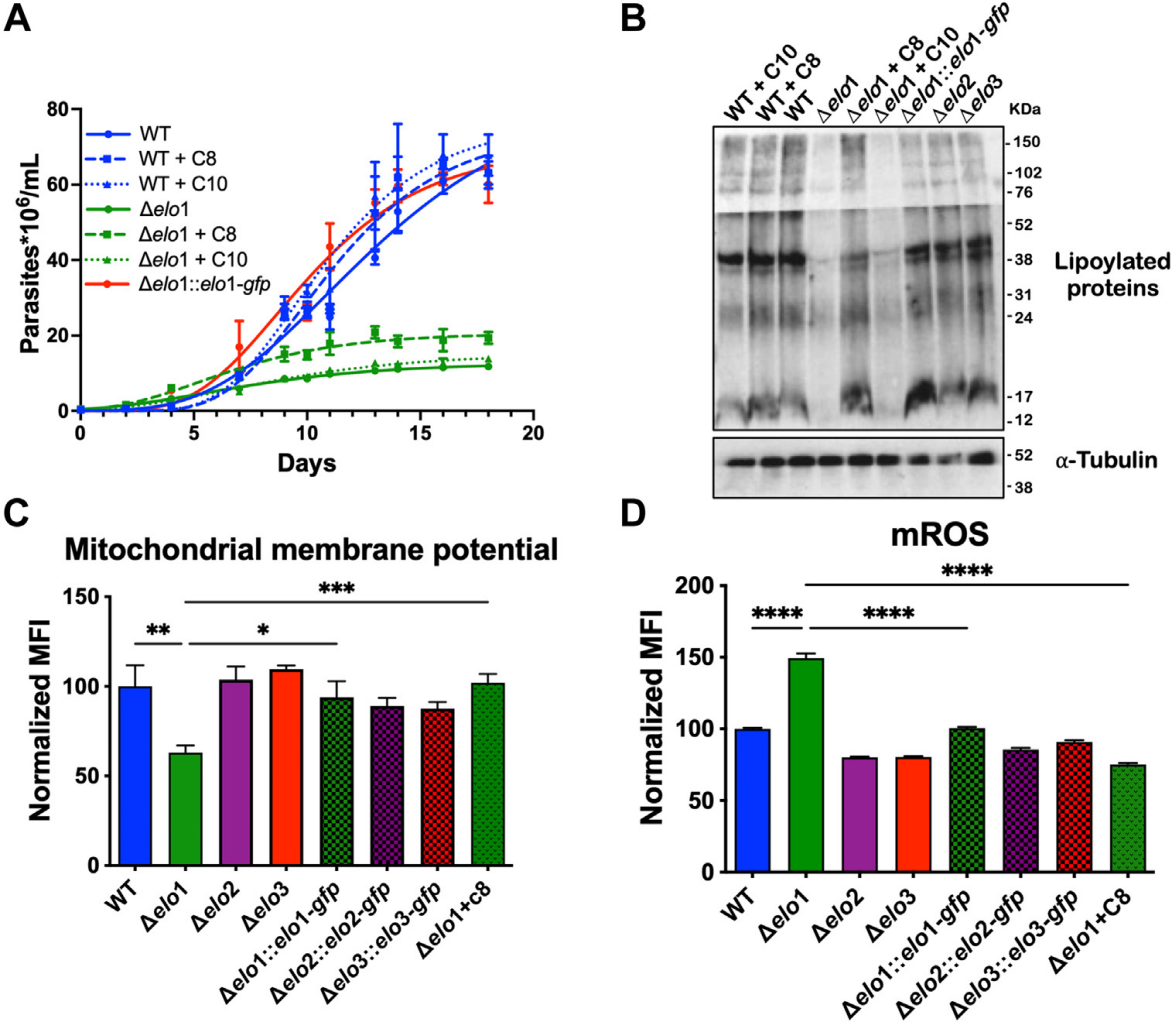
**Protein lipoylation is impaired in Δ*elo*1 mutants.***A*, growth curves of WT, Δ*elo*1, and Δ*elo*1::*elo*1 EPI grown in LIT media with and without supplementation with 35 μM C8:0 or C10:0. The mean and SD calculated for parasite density (parasites × 10^6^/ml medium; *y*-axis) for three biological replicates against time (days; *x*-axis) is plotted. *B*, Western blot of *T. cruzi* EPI lysates harvested on the final day of the growth curve (*A*) in the 35 μM of C8 or C10 FA and probed with antibodies to lipoic acid (*top*). Blots were stripped and reprobed with an anti-α-tubulin antibody (*bottom*). *C*, mitochondrial membrane potential in *T. cruzi* EPI lines after incubation with 35 μM of C8 measured by TMRE staining and flow cytometric analysis. *D*, mitochondrial reactive oxygen species after incubation with 35 μM of C8, measured by MitoSOX staining and analyzed by flow cytometry. One-way ANOVA with Dunnett's multiple comparisons test was applied (∗*p* < 0.05) in all analyses. FA, fatty acid; LIT, liver infusion tryptose.

The metabolic tracing experiments above confirmed that ELO1-deficient *T. cruzi* EPI forms are defective in their ability to elongate butyric acid (C4:0). To explore the possibility that the loss of FA products or intermediates generated by ELO1 (*e.g.*, C8:0 and C10:0) might underlie growth and mitochondrial phenotypes observed in Δ*elo1* mutant EPI, growth curve experiments were conducted in complete LIT medium supplemented, or not, with 35 μM octanoic acid (C8:0) or decanoic acid (C10:0). C10:0 supplementation failed to alter the growth of WT or Δ*elo1* EPI, whereas C8:0 exerted a small but significant growth-enhancing effect on this mutant (Fig. 7*A*). As octanoic acid (C8:0) is used to synthesize lipoic acid, a cofactor needed for the stabilization and activity of mitochondrial enzymes such as pyruvate dehydrogenase (PDH) and α-ketoglutarate dehydrogenase (KDH), key regulators of oxidative metabolism in mitochondria (39), we sought to determine whether protein lipoylation levels were altered in Δ*elo1* mutant EPI. As a covalent post-translational modification, lipoylation of the E2 subunits of these enzymes is readily detected by Western blot using an antibody that recognizes lipoic acid (25). This approach detected several bands corresponding to the E2 subunits of lipoylated proteins in protein lysates prepared from WT *T. cruzi* EPI, as previously reported (25). The protein lipoylation signal, normalized to tubulin, was significantly reduced in Δ*elo1* mutant EPI (52%) as compared to WT, Δ*elo2* and Δ*elo3* parasites (Fig. 7*B*). Reduced protein lipoylation observed in the Δ*elo*1 mutant was fully restored in the genetically complemented Δ*elo1::elo1-gfp* EPI line (Fig. 7*B*). Moreover, supplementation of the medium with 35 μM C8:0 resulted in partial restoration of protein lipoylation in the Δ*elo1* mutant. In contrast supplemental C10:0 had no detectable effect on lipoylation in the Δ*elo1* mutant or WT (Fig. 7*B*). We further demonstrate that media supplementation with C8:0 completely restores mitochondrial membrane potential and mROS similar to restoration achieved with genetic complementation (Fig. 7, *C* and *D*). These results provide pioneering evidence of a functional link between ELO1 activity and protein lipoylation in trypanosomatids.

## Discussion

LCFA play a critical role in the growth and developmental cycle of *T. cruzi*, where 16- and 18-carbon species are the most abundant FA in this organism (12, 27, 29, 30). Like many cells, *T. cruzi* can synthesize LCFA *de novo* and scavenge FA from the environment (12, 35). Bulk FA synthesis, *via* the ELO system, takes place at the cytoplasmic face of the ER and is physically and biochemically distinct from the mitochondrial FAS-II system (12, 13). In this study, we generated genetic mutants to examine the role(s) of the first three FA ELOs in the *T. cruzi* ELO system, ELO1, ELO2, and ELO3, predicted to be responsible for the processive generation of C16:0 and C18:0 from C4:0 (butyryl-CoA) (13). Phenotypic analysis of individual Δ*elo* mutants and their genetically complemented lines has provided novel perspectives on these ELOs in lipidome homeostasis, mitochondrial metabolism, and parasite growth.

Our metabolic tracing studies confirmed that WT *T. cruzi* EPI possess a functional ELO system that can generate LCFA from C4:0 in a processive manner. The short-chain FA ELO, ELO1, is required to elongate C4:0 precursors, as this capacity was abolished in Δ*elo1* mutant *T. cruzi* EPI and restored with genetic complementation. Despite its role in generating LCFA from C4:0, ELO1 is not required for global lipidome maintenance in *T. cruzi* EPI. ELO1-deficient parasites still produce LCFA, presumably *via* the actions of ELO2 and ELO3, and these parasites display minimal changes in their phospholipid and neutral lipid profiles. These findings argue that the operation of a processive ELO system (*e.g.*, from C4:0 to C18:0) is not essential for lipidome maintenance in *T. cruzi* EPI and predict similar outcomes for related trypanosomatids, for which the global impact of ELO KO has not been determined (13).

In contrast to ELO1, both ELO2 and ELO3 exerted evident influence over the lipidome in *T. cruzi* EPI. Marked changes in the lipidome profiles of both Δ*elo3* and Δ*elo2* mutant EPI were consistent with the loss of specific ELO activities. For instance, complex lipids (*e.g.*, phospholipids, sphingolipids, neutral lipids) containing C16 and C18 FA moieties were significantly diminished in Δelo*3* and Δ*elo2* EPI. In contrast, lipids containing the uncommonly used C10, C12, and C14 fatty acyl species were elevated in these mutants. These global lipidomic changes were attributable to the loss of specific ELO function as a reversion to WT profiles was evident in the respective genetically complemented lines. Notably, global lipidomic changes were observed in Δ*elo3* and Δ*elo2* mutant EPI despite the continuous passage of parasites in a medium containing serum. Thus, serum lipids and exogenous FAs appear insufficient to compensate for the loss of ELO2 or ELO3 function and the resultant lipidomic changes occurring in these mutants. Metabolic tracing studies demonstrate the ability of Δ*elo* mutants to incorporate ^14^C-labeled LCFA precursors into phospholipid and neutral lipid pools at levels similar to WT EPI. However, we cannot exclude the possibility that the mutants exhibit quantitative differences in importing or utilizing exogenous FA.

One of the unexpected findings of this study was the inverse relationship between global lipidome perturbation and the growth of *T. cruzi* Δ*elo* mutants. ELO1-deficient parasites exhibited severe growth impairment with relatively few lipidomic changes, whereas Δ*elo2* and Δ*elo3* mutants showed little or no growth impairment, respectively, despite evident lipidome perturbation. Notably, media supplementation with exogenous LCFA failed to rescue growth inhibition observed in Δ*elo1* or Δ*elo2* mutant EPI, despite evidence of uptake and incorporation of exogenous FA into parasite neutral and polar lipids. Coculturing the Δ*elo1* mutant with WT parasites also failed to improve parasite growth, diminishing the likelihood that defects in production or release of growth stimulating factors (if any exist in this organism) are responsible for growth impairment of ELO1-deficient *T. cruzi* EPI. Combined, these observations indicate that the severe growth defect associated with the loss of ELO1 expression is unlikely to be related to the failure of this mutant to generate, or to acquire, LCFA.

Accordingly, changes in polar metabolite profiles in log-phase Δ*elo* mutant EPI were better aligned with the growth phenotypes of these mutants. Specifically, the most significant metabolic perturbation occurred in the Δ*elo1* mutant (impaired growth) while minimal changes occurred in the Δ*elo3* mutant (no growth impairment). Of the 74 metabolites that exhibited significant differences in abundance in Δ*elo1* EPI, the most striking was malonyl-CoA, which increased 50-fold over WT in the Δ*elo1* mutant and was restored to near WT levels in genetically complemented parasites. Given the pivotal role of malonyl-CoA in FA and energy metabolism, the accumulation of this metabolite is expected to trigger dramatic metabolic changes in the parasite. Malonyl-CoA is consumed in the synthesis of FA (in both the ELO and mitochondrial FAS-II systems), and conditions that lead to high levels of malonyl-CoA are known to suppress FA entry into mitochondria through the direct inhibition of carnitine palmitoyltransferase 1 (40, 41, 42). Consistent with the anticipated metabolic consequences of malonyl-CoA accumulation, FAO was significantly decreased in Δ*elo1* mutant EPI and restored to WT levels in genetically complemented Δ*elo1::elo1-gfp* parasites. Why malonyl-CoA accumulates considerably in ELO1-deficient parasites, but not in the other ELO KOs, is perplexing, given that this two-carbon donor is utilized to elongate fatty acyl-CoA substrates by all ELO enzymes. One possibility is that the ELO1-dependent FA elongation cycle functions at a higher rate than the combined rates of all other ELOs, leading to excess malonyl-CoA in the absence of ELO1. Alternatively, excess malonyl-CoA may be symptomatic of reduced mitochondrial FAS-II activity in the Δ*elo1* mutant.

**Figure 8:**
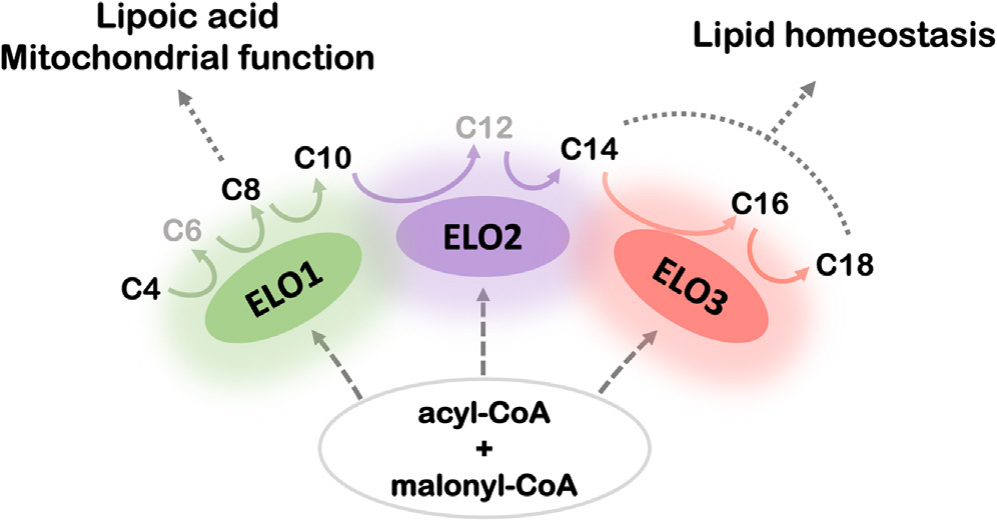
**ELO pathway model.** Schematic of the ELO pathway showing the contribution of ELO enzymes to lipid homeostasis. An acyl-CoA and malonyl-CoA are the substrates for the ELO enzymes. Main substrates and products are indicated in *black* and intermediate products are indicated in *gray*. Glow indicates function overlapping between the ELO enzymes. *Solid-colored arrows* match the main ELO enzymes’ activities. ELO1 elongates C4 up to C8, which can be presumably used for lipoic acid synthesis or elongated to C10. ELO2 elongates C10 up to C14, which will be taken up by ELO3 and elongated up to C16 or C18 and used for complex lipid synthesis. Exogenous FA can be incorporated at any level of the ELO pathway for further elongation. FA, fatty acid.

Indeed, there are indicators of mitochondrial dysfunction in ELO1-deficient *T. cruzi* EPI, including reduced mitochondrial membrane potential, increased ROS production, and significant alteration in the levels of metabolites related to energy and redox metabolism. In addition, the finding that ELO1 expression impacts protein lipoylation, a rare but highly conserved posttranslational modification, connects this short-chain FA ELO to mitochondrial metabolism. The activity of four mitochondrial enzymes (PDH and α-KDH, branched-chain α-ketoacid dehydrogenase and the glycine cleavage system) requires the post-translational attachment of lipoic acid (6,8-dithiooctanoic acid) which is derived from octanoic acid (C8:0) (25, 39). We have shown that protein lipoylation is significantly diminished in Δ*elo1* EPI and partially restored by the addition of C8:0, an intermediate product of ELO1-dependent FA elongation. Reduced lipoylation of these key mitochondrial enzymes is expected to impact enzymatic function and affect mitochondrial metabolism. For example, functioning of the TCA cycle would be impaired with reduced PDH and KDH activities. The accumulation of α-ketoglutarate and reduction of succinate in the Δ*elo1* mutant are consistent with such a prediction. While octanoic acid used for the synthesis of lipoic acid is assumed to be produced by FAS-II (25, 39, 43), our results reveal a previously unrecognized link between microsomal ELO1 activity and protein lipoylation in *T. cruzi* (Fig. 8). At this point, it is unknown whether octanoic acid generated by ELO1 directly contributes to lipoic acid pools in the parasite, or whether the global metabolic perturbation and mitochondrial dysfunction, demonstrated in ELO1-deficient parasites, impacts FAS-II activity indirectly. As supplemental C8:0 rescues mitochondrial dysfunction but only provides a minor boost to growth of Δ*elo1* EPI, our data suggest that growth impairment accompanying loss of ELO1 function is likely the culmination of multiple metabolic and cellular changes.

This study, which represents the first functional investigation of the FA ELOs, ELO’s 1-3 in *T. cruzi,* reveals previously unrecognized complexity in the trypanosomatid ELO pathway. Consistent with current knowledge of the *T. brucei* ELOs (26, 44), the *T. cruzi* ELO’s 1-3 can operate sequentially to elongate four-carbon FA substrate to produce C16:0 and C18:0, where some functional overlap between the enzymes is indicated (Fig. 8). As a modular system, the individual ELO’s can act independently to contribute to different cellular processes, as revealed here. While the activities of ELO2 and ELO3 have a clear impact on the global lipidome of *T. cruzi* epimastigotes, ELO1 appears to contribute little to the synthesis of membrane phospholipids or maintenance of the lipidome profile in this parasite. Instead, ELO1 activity is required to promote mitochondrial function and normal growth of *T. cruzi* EPI. Evidence points to the role of ELO1 in providing the FA precursor, octanoic acid, for the synthesis of lipoic acid, a cofactor required for the activity of key mitochondrial enzymes. Whether causal or symptomatic of other unidentified metabolic/physiologic changes in the parasite that occur with loss of ELO1, the link between ELO1 activity and mitochondrion is a novel finding that highlights the trypanosomatid ELO pathway as a critical metabolic regulator.

## Experimental procedures

### Reagents

FAs were purchased from Sigma-Aldrich and 100x (3.5 mM) stocks of butyric acid, decanoic acid, myristic acid, palmitic acid, and stearic acid were prepared by dissolving each FA in 2 ml ethanol at 60 °C, followed by dilution in prewarmed (37 °C) PBS containing 100 mg/ml bovine serum albumin (BSA). Oleic acid solution was purchased suitable for cell culture. All radiolabeled compounds were purchased from American Radiolabeled Chemicals Inc.

### Trypanosoma cruzi maintenance and growth curves

Tulahuén strain *T. cruzi* (ATCC PRA-33) EPIs were grown axenically at 27 °C in LIT, supplemented with 10% fetal bovine serum, 100 U/ml penicillin, 10 μg/ml streptomycin, and maintained in log-phase by weekly passage. For comparative growth analysis, log-phase *T. cruzi* EPIs were diluted to a density of 1 × 10 (6) parasites/ml in LIT and incubated at 27 °C for 15 to 20 days. Twenty-five microliters samples were collected every 24 h, fixed in 75 μl of 2% paraformaldehyde (PFA)/PBS, and stored at 4 °C. Once all samples were collected, the number of parasites present in 100 μl was counted using a MACSQuant VYB Flow Cytometer. For the biochemical supplementation experiments, LIT was supplemented with FAs at a final concentration of 35 μM and parasite growth curves were derived. For coculturing Δ*elo* mutants with WT parasites, parasites were seeded in transwell microplates, with a 0.4-μm pore size, to allow media exchange between cultures. All growth curves were performed in three independent experimental rounds, with biological triplicates in each round.

### CRISPR/Cas9-mediated disruption of ELO genes in *T. cruzi*

Gene disruption was accomplished using a homology-directed repair, Cas9-mediated system modified from Lander *et al.* (45, 46). Two guide RNA targeting sequences were designed for each target gene using the EuPaGDT system (47). Mutations to the specificity of guides in pTREX-n-Cas9 were performed using a Q5 mutagenesis kit (NEB). Guides targeting the ELO genes were inserted using the primers described in Table S5: ELO 1 (*elo1*, TcCLB.506661.30, g-*elo*1-1 and g-*elo*1-2 for g163 and g-*elo*1-3, and g-e*lo*1-4 for g151). ELO 2 (*elo2*, TcCLB.506661.20), g-*elo*2-1 and g-*elo*2-2 for g100, and g-*elo*2-3 and g-*elo*2-3 for g92. ELO 3 (*elo3*, TcCLB.506661.10), g-*elo*3-1 and g-*elo*3- for g116 and g-*elo*3-3, and g-*elo*3-4 for g132. Donor DNA containing the drug resistance cassette was generated by PCR using ultramers with 100-bp homology to regions flanking the predicted Cas9 cut site and an in-frame P2A ribosomal skip peptide. Ultramer primers (Table S5): u-*elo*1-1 and u-*elo*1-2 were used for *elo1*, u-*elo*2-1, and u-*elo*2-2 for *elo2* and u-*elo*3-1, and u-*elo*3-2 for *elo3*. Parasites were transfected simultaneously with pTREX-Cas9-guide RNA plasmids and donor DNA.

### Parasite transfection

Log-phase *T. cruzi* epimastigotes were transfected with plasmid DNA using an Amaxa nucleofector, program U-33 in Tb-BSF (48). Transfected parasites were allowed to recover for 24 h and subsequently cloned in the presence of a drug corresponding to the resistance marker in the donor DNA. Clones were screened by PCR using the following primers: s-Elo1-1 and s-Elo1-2 for *elo1*, s-Elo2-1 and s-Elo2-2 for *elo2*, and s-Elo3-1 and s-Elo3-2 for *elo3* (Table S5).

### Southern blot

Southern blots were accomplished following the specifications of the ECL Direct Nucleic Acid Labeling and Detection System (GE Healthcare). Briefly, genomic DNA was digested with PstI and EcoRI (*elo1*) or DraI and EcoRI (*elo2* and *elo3*), separated on an agarose gel, and blotted into a nitrocellulose membrane. Membranes were incubated with specific probes and developed using ECL reagent. Probes were generated by PCR using the following primers listed in Table S5: p-Elo1-1 and p-Elo1-2 for *elo1*, p-Elo2-1 and p-Elo2-2 for *elo2* and p-Elo3-1, and p-Elo3-2 for *elo3*.

### Genetic complementation

*Elo* genes were amplified by PCR using the following primers (Table S5): f-Elo1-1 and f-Elo1-2 for *elo1*, f-Elo2-1, and f-Elo2-2 for *elo2*, and f-Elo3-1 and f-Elo3-2 *elo3*. PCR products were cloned into the pTREX-GFP plasmids between the Not1 and Spe1 restriction sites. Log-phase EPIs were transfected with pTREX-GFP-Elo plasmids. Transfected EPIs were allowed to recover for 24 h and, subsequently, cloned in the presence of a drug corresponding to the resistance marker in the plasmid.

### Metabolic tracing and TCL

*T. cruzi* EPIs (1 × 10 (8)) were incubated with 0.3 μCi/ml ^[1-14]^C-labeled FAs for 18 h, and total lipids were extracted with chloroform:methanol (2:1, v/v) and dried under N_2_ gas. Modified Folch’s partition, using chloroform:methanol:water (4:2:1.5 v/v/v/) (49), was carried out on two-thirds of total lipids, and neutral and polar lipids were resolved by TLC using silica-gel matrix. FAMEs were generated from one-third of the remaining lipids by incubating with 1 M KOH in methanol for 2 h at 68 °C. Folch’s partition was carried out on the generated FAMEs and FAs were resolved by RP-TLC, using a silica-gel 60 RP-18 matrix (Sigma-Aldrich). Mobile phases used were as follows: acetone:methanol:acetic acid:chloroform:water (15:13:12:40:8, v/v/v/v/v) for polar lipids, hexane: diethyl-ether: acetic acid (80:20:3, v/v/v) for neutral lipids, and chloroform:methanol:water (5:15:1, v/v/v) for FA length chain analysis on RP-TLC. The equivalent of 2 × 10 (7) parasites was spotted per lane. Labeled lipids were detected by phosphor-imaging (Typhoon FLA 7000, GE).

### Protein quantification and lipid extraction

EPIs (2 × 10^8^) were resuspended in 500 μl ice-cold PBS and a small aliquot (30 μl) was removed for protein quantification (Pierce BCA Protein Assay Kit; Thermo Fisher Scientific). For lipid quantification, EquiSPLASH mix (Avanti Polar Lipids) deuterated standards were spiked into the first extraction solvent mixture (chloroform:methanol:PBS, 1:2:0.8, v/v/v). For every 50 μg of protein, 1 μl EquiSPLASH mix (Avanti Polar Lipids) was added before cell extraction. Samples were vortexed vigorously for 1 min and centrifuged at 1200*g* for 10 min, with the resulting supernatant transferred to a fresh Supelco PTFE vial. Remaining parasite pellets were further subjected to three sequential extractions with chloroform:methanol (2:1, v/v), pooled, and dried under constant N_2_ gas. Finally, lipids were separated by modified Folch’s partition (49). Folch’s lower phase was separated, pooled, dried under N_2_ gas, and stored at −20 °C. Folch’s lower phase were redissolved in 100 μl chloroform:methanol (1:2, v/v) for UHPLC-LC-MS/MS analysis. Five microliters of each sample were combined into a single pool for quality control (QC).

### Liquid chromatography-high resolution tandem mass spectrometry

Ten microliters of each biological sample were run in two technical replicates, with QC samples processed a total of four times throughout each positive and negative data acquisition by reverse phase Kinetex C18 EVO 2.6 μm, 100 Å, 150 × 2.1 mm (Phenomenex) column attached to a Dionex Ultimate 3000 UHPLC (Thermo Fisher Scientific). The column was heated to 45 °C with of 0.5 ml/min flow rate throughout the entire run. Equilibrated with 30% solvent A (50% acetonitrile, 10 mM ammonium formate, 0.1% formic acid), 70% solvent B (88% isopropanol, 10% acetonitrile, 2 mM ammonium formate, 0.02% formic acid) and was maintained for 3 min, raised to 43% solvent B over 3 min, 45% over 0.2 min, 65% over 9.8 min, 85% over 6 min, 100% over 2 min where the plateau was maintained for 5 min dropped to 30% solvent B over 0.1, and re-equilibrated for 2.9 min before next sample injection. Data was acquired from eluting metabolites on a Q-Exactive Plus Hybrid Quadrupole Orbitrap Mass Spectrometer (Thermo Fisher Scientific) equipped with a HESI-II source. Before starting the acquisition, 10 μl of blank chloroform:methanol (1:2, v/v) were processed; manual selection of the top ions for both positive and negative ion mode were imported into an exclusion list (50). Individual samples and their respective technical replicates were processed in Full Scan at 140,000 resolution (51, 52, 53). Pooled QC samples were processed in top 8 data-dependent MS (2) mode at 70,000 Full MS with 5e (5) automatic gain control target, at a scan range of 200 to 1450 *m/z* 35,000; and MS (2) at 35,000 resolutions with 1e (6) automatic gain control target (50, 52, 53).

### Lipidomic data analysis, statistics, and graphics generation

Raw files were processed utilizing LipidMatch flow in both positive and negative on modes (51, 52, 53). Processed samples were normalized by each lipid species from the EquiSPLASH mix with the highest abundance area and their calculated corresponding spiked abundance values in pmol. If lipid species were not identified with their respective deuterated standard species, the lipid was normalized to *lyso*-phosphatidylcholine 18:1-d7. Normalized lipids were utilized to generate heatmaps and PCA by uploading to Metaboanalyst 4.0, submitted as concentrations, filtered by the relative SD, normalized to a WT as a reference sample, log-transformed, and auto-scaled as per the workflow of MetaboAnalyst (54). PCA was generated in addition to generated heatmap specifics including a distance measurement set to Euclidean, clustering average, top 50 PLS-DA VIP. Bar graphs were generated by averaging the raw data values in Prism (GraphPad v8.3.1).

### Metabolomics

EPIs (2 × 10^8^) in the exponential growth phase were pelleted by centrifugation at 1200*g* for 5 min, resuspended in 4 ml of methanol at −80 °C, and incubated for 30 min at −80 °C. Samples were centrifuged at full speed for 5 min and the supernatant was transferred to a new tube. The extraction procedure was repeated twice and the resulting supernatants were combined and dried under N_2_ gas. Dried samples were resuspended in 20 μl of HPLC water before running on a 5500 QTRAP hybrid triple quadrupole mass spectrometer as described (55).

### Cell cycle analysis by flow cytometry

The cell cycle was analyzed as previously reported (56). Briefly, exponentially growing *T. cruzi* epimastigotes were fixed on ice in 4% PFA/PBS for 20 min. After fixation parasites were centrifuged at 4000*g* for 10 min; the resulting pellet was resuspended in PBS. Immediately before the acquisition, parasites were pelleted at 1500*g* for 10 min and resuspended in a 0.1% Triton X-100/PBS permeabilization solution containing 10 ng/ml 4′,6-diamidino-2-phenylindole (DAPI) (Sigma-Aldrich) for a minimum of 30 min on ice and analyzed using a MACSQuant VYB flow cytometer. Parasites were identified based on size and DAPI staining. Proliferation modeling based on signal intensity was generated using FlowJo (Tree Star) proliferation software (https://www.flowjo.com). Greater than 10,000 events in the final EPI gate were acquired for each sample. Cell cycle analysis was performed in two independent experimental rounds, with biological triplicates in each round.

### Immunofluorescence microscopy

Parasites were fixed in 1% PFA-PBS for 10 min, spotted onto poly-L-lysine coated slides and allowed to adhere for 30 min. Adhered parasites were washed with PBS and permeabilized with 0.1% Triton X-100-PBS for 10 min, rewashed again with PBS, and blocked with 3% BSA (Sigma-Aldrich) in PBS for 1 h. Then, parasites were incubated with anti-BIP antibodies in 1% BSA in PBS for 1 h (a generous gift from Dr J. D. Bangs, U. Buffalo) and washed three times with PBS before incubation for 1 h with goat anti-rabbit IgG secondary antibody Alexa fluor 594 (Thermo Fisher Scientific), and 100 ng/ml DAPI (Sigma-Aldrich). Finally, parasites were washed with PBS and mounted with ProLong Antifade (Thermo Fisher Scientific). EPIs were analyzed using a Yokogawa CSU-X1 spinning disk confocal system paired with a Nikon Ti-E inverted microscope equipped with an iXon Ultra 888 EMCCD camera. All images were acquired with the 100× objective and image processing was completed using ImageJ Fiji (https://fiji.sc) (57).

### Mitochondrial membrane potential

Mitochondrial membrane potential was measured using TMRE Mitochondrial membrane Potential Assay Kit (Abcam), according to the manufacturer’s instructions. Fluorescence intensity was measured by flow cytometry (MACSQuant VYB) for >10,000 events in the final EPI gate were acquired for each sample. Each experiment was carried out in triplicate in at least two independent experiments. Fluorescence intensity was analyzed using FlowJo software (version 10.7.2).

### Superoxide determination

Mitochondrial superoxide determination was accomplished following the specifications of MitoSox Red mitochondrial superoxide indicator (Invitrogen). Briefly, treated or untreated EPI (5 × 10^6^ cells) in exponential growth were collected and resuspended in 1 ml of LIT media and 100 μl were settled in 96-well plate. Hundred microliteres of MitoSox staining solution or LIT was added to the samples and incubated for 60 min at 27 °C. Fluorescence intensity was acquired by flow cytometry. Over 10,000 events in the final EPI gate were acquired for each sample. Each experiment was carried out as biological triplicates and at least two experimental rounds. Fluorescence intensity was analyzed with FlowJo 10.7.2.

### FAO assay

FAOβ assay was performed, as described (58). Briefly, EPI cells (1 × 10^8^ cells) in exponential growth were centrifuged at 1200*g* for 5 min and resuspended in 1 ml of PBS with 10 μCi of ^3^H-palmitate and 0.25 mM carnitine or without supplementation. Hundred microliteres samples were collected after 4 h of incubation and centrifuged at 3000*g* for 5 min. The supernatant was transferred to a new tube and 100 μl of 10% TCA was added and incubated for 15 min. The supernatant was mixed with 5% TCA and 10% BSA, incubated at RT for 15 min, and extracted with chloroform:methanol (2:1, v/v) and 300 μl of a 2 M potassium chloride: 2 M hydrochloride mix was added. The supernatant was mixed with 5 ml of Ultima Gold XR (PerkinElmer). The count-per-minute was determined for 5 min for each sample. The count-per-minute with a ^3^H-labeled medium was used as background to correct all samples. Final results were obtained after normalization using the protein concentration. FAO assays were performed in three independent experimental rounds, with biological triplicates in each round.

### Western blot

EPI pellets were washed twice with PBS and resuspended in lysis buffer (0.5% Nonidet P-40, 500 mM NaCl, 5 mM EDTA, 1 mM DTT, 50 mM Tris-Base, 0.4% SDS, pH 7.4) at 1 × 10^6^ parasites/μl. Samples were sonicated using 3 pulses of 30 s at 100% amplitude (Q700 sonicator, QSONICA), with 15 s breaks between to rest the tubes on the ice. Samples were centrifuged at 16,000*g* for 20 min and the supernatant was collected. Equivalent to 1 × 10^6^ parasites were resolved by electrophoresis on a Mini-Protean TGX precast gel (Bio-Rad). Lipoylated proteins were detected with rabbit antilipoic acid antibody (ab 58,724, Abcam). Bound antibodies were detected with horseradish peroxidase-linked anti-rabbit IgG (GE Healthcare) and ECL Prime Western Blotting Detection Kit (GE Healthcare). The loading control was performed with mouse anti-α-tubulin (Sigma-Aldrich) and bounded antibodies were detected with horseradish peroxidase-linked anti-mouse IgG (GE Healthcare). Quantification was performed after normalization of lipoic acid signal to tubulin, using FIJI (57). Each experiment was carried out in at least three independent experiments.

## Data availability

All data are contained within the article.

## Supporting information

This article contains supporting information.

## Conflict of interest

The authors declare that they have no conflicts of interest with the contents of this article.
