# Supplementary material for: Fatty acid elongases 1-3 have distinct roles in mitochondrial function, growth, and lipid homeostasis in Trypanosoma cruzi
Source: J Biol Chem. 2023 Apr 13;299(6):104715. doi: 10.1016/j.jbc.2023.104715 (PMC10203773; doi:10.1016/j.jbc.2023.104715)
Supplement: Supporting Table S5 [file mmc5.docx]

**Table S5. Primers used in the generation of knockouts and transgenic *T. cruzi* lines**

| **Target** |  | **Primers 5’-3'** |
| --- | --- | --- |
| **pTREX-Cas9 modification** | | |
| pTREX-Cas9 | pTREX-1  pTREx-2 | Fw: CCCAAAAAGAAAAGGAAGGTTGATTAGAAGCTTATCGATACCGTCGAC  Rv: GTCCTCGACTTTTCGCTTCTTTTTCGGGTCGCCTCCCAGCTGAGA |
|  | | |
| **Guides** | | |
| *elo*1-g136 | g- *elo*1-1  g- *elo*1-2 | Fw: CGGGCCGCGAGTTTTAGAGCTAGAAATAGC  Rv: TAATTTTCGCGGATCCACTAGAACTCTTG |
| *elo*1-g151 | g- *elo*1-3  g- *elo*1-4 | Fw: AACAAATGCGGTTTTAGAGCTAGAAATAGC  Rv: TTCACTGGACGGATCCACTAGAACTCTTG |
| *elo*2-g100 | g- *elo*2-1  g- *elo*2-2 | Fw: ACCGAGGCTCGTTTTAGAGCTAGAAATAGC  Rv: TATCTGGCACGGATCCACTAGAACTCTTG |
| *elo*2-g92 | g- *elo*2-3  g- *elo*2-4 | Fw: TGTGTGGGAAAGTTTTAGAGCTAGAAATAGC  Rv: AGTGCCAGATGGATCCACTAGAACTCTTG |
| *elo*3-116 | g- *elo*3-1  g- *elo*3-2 | Fw: CCGCAACAGCGTTTTAGAGCTAGAAATAGC  Rv: TGGTGCTGTAGGATCCACTAGAACTCTTG |
| *elo*3-132 | g- *elo*3-3  g- *elo*3-4 | Fw: GAAAACGTCAGTTTTAGAGCTAGAAATAGC  Rv: AGGTACGTACGGATCCACTAGAACTCTTG |
|  | | |
| **Ultramers** | | |
| *elo*1 | u- *elo*1-1  u- *elo*1-2 | Fw:TCAAGAAAATGGAATTTGTGCAGAATTGGGACGGTTACGCAGTCCGCGATTGGATGATTCGGAATGTGGATGTGGTGGGGTATATTTCTGGCATTGCTACTAACTTCAGCCTGCT  Rv:GAAGACAGAGAGGGATAAATTCCACAACACCATGGCACGACGTACCGCCTTGGACCCACTGCCACCTGCAACACCTTGCCTTGCGGGTGCTACAGATGTTCTAGGCGGCCGCTCTAGAACTAGTGGA |
| *elo*2 | u- *elo*2-1  u- *elo*2-2 | Fw:aaaaaagtaatgtttccgtacgttgaagactacgacggctatgccgttaagagactgatgctagagaacgtggacgttcttggcGCTACTAACTTCAGCCTGCT  Rv:gaacgccgacaacaggaggttccagacaatgatggcgtagcgaagaagccccgagttgctcacacggttcgtttccccattcaggcgcctcaccacagcaCTAggcggccgctctagaactagtgGA |
| *elo*3 | u- *elo*3-1  u- *elo*3-2 | Fw:ttgggaaatggcaattgcatggatggactcgtacacccgatgggcggcggacttccgtggcgaacatctacggagttggatgcgtgatcacacggaggtgGCTACTAACTTCAGCCTGCT  Rv:ccacagtcgcggtaaacagtagtacgccccgcatatagagaacactgtgagcagcagattccataacatgttcagaaaacgcagttttataggattgcggCTAggcggccgctctagaactagtgGA |
|  | | |
| **Screening** | | |
| *elo*1 | s- *elo*1-1  s- *elo*1-2 | Fw: agagagggagagagaggccatt  Rv: tcctcttctccttttccccagc |
| *elo*2 | s- *elo*2-1  s- *elo*2-2 | Fw: gtgcctgtacgtctttttcggg  Rv: tcccctctatgtgcatgaacac |
| *elo*3 | s- *elo*3-1  s- *elo*3-2 | Fw: gagaaccctgcgtctgtgtgta  Rv: ttgctatcgtccttccgttttt |
|  | | |
| **Cloning as GFP fusion** | | |
| *elo*1 | f- *elo*1-1  f- *elo*1-2 | Fw: ataGCGGCCGCatggaatttgtgcagaattggg  Rv: tatACTAGTgctagactttttcaaggagcc |
| *elo*2 | f- *elo*2-1  f- *elo*2-2 | Fw: tatGCGGCCGCatgtttccgtacgttgaagac  Rv: tatACTAGTcaccttcttccctgcgg |
| *elo*3 | f- *elo*3-1  f- *elo*3-2 | Fw: catGCGGCCGCatggcaattgcatggatgg  Rv: atcACTAGTctttccttttttctccccg |
|  | | |
| **Southern probes** | | |
| *elo*1 | p- *elo*1-1  p- *elo*1-2 | Fw: acacgacggatgtagggttctg  Rv: cctgcacacctcagctagactt |
| *elo*2 | p- *elo*2-1  p- *elo*2-2 | Fw: cggggttctggatcggtatgtt  Rv: gcaagtcacaccttcttccctg |
| *elo*3 | p- *elo*3-1  p- *elo*3-2 | Fw: accaaaattgccaggcagcttt  Rv: acatcaggagtcccattcgcaa |
